# Editorial: Neuroscience-driven visual representation

**DOI:** 10.3389/fnins.2023.1345688

**Published:** 2023-12-19

**Authors:** Teng Li, Qieshi Zhang, Fudong Nian, Caifeng Shan

**Affiliations:** ^1^Information Materials and Intelligent Sensing Laboratory of Anhui Province, Anhui University, Hefei, China; ^2^Hefei Comprehensive National Science Center, Institute of Artificial Intelligence, Hefei, China; ^3^Shenzhen Institute of Advanced Technology, Chinese Academy of Sciences (CAS), Shenzhen, Guangdong, China; ^4^School of Advanced Manufacturing Engineering, Hefei University, Hefei, Anhui, China; ^5^College of Electrical Engineering and Automation, Shandong University of Science and Technology, Qingdao, China; ^6^School of Intelligence Science and Technology, Nanjing University, Nanjing, China

**Keywords:** visual representation, computer vision, neuroscience, learning, deep neural networks

Visual representation learning seeks to mimic the human visual system using deep neural networks, enabling machines to interpret digital images and video for diverse applications from manufacturing to energy. However, major gaps remain compared to biological vision, and most representation learning methods do not sufficiently incorporate neuroscientific and psychological principles. Key open questions persist around designing optimized architectures to extract meaningful representations from complex 2D or 3D scenes containing numerous heterogeneous, unlabeled examples.

While deep learning has achieved state-of-the-art results across various vision tasks like classification, detection and segmentation, core challenges in representation learning need to be tackled to reach human-level visual understanding. For instance, handling unlabeled, unstructured data and generalizing learned patterns to novel datasets continue to pose difficulties. Furthermore, lack of model interpretability is an issue that integration of biological approaches could help address.

This research area aims to advance visual representation learning through synergistic fusion of deep neural networks with psychological and neuroscientific concepts. By providing a platform to exchange cutting-edge techniques spanning both data-driven and theory-driven disciplines, impactful progress can be made toward biomimetic visual systems. Realizing more efficient, generalizable, and explainable visual learning has the potential to profoundly transform capabilities in scientific imaging, manufacturing, transport and healthcare.

Enhanced analysis of facial imagery for health assessment. Building on advanced computer vision techniques, Li et al. present a facial analysis methodology using convolutional neural networks (CNNs) to detect depression. They introduce innovations including multi-head attention modules and region-specific tuning to improve CNN sensitivity in analyzing different facial areas tied to depression. With further research, such AI-based systems could assist in mental health evaluation and screening.

Multi-constraint modeling for 3D shape reconstruction. Reconstructing 3D structure from 2D image sequences is an important but challenging computer vision task. Chen X. et al. put forth a multi-constraint estimation algorithm that first extracts shape bases via sparse coding, then estimates 3D geometry through a penalized least-squares model incorporating orthogonal and similarity constraints. Experiments demonstrated higher accuracy compared to existing methods, showing the value of fusing multiple constraints.

Automated defect recognition for semiconductor quality control. As discussed by Chen Y. et al., precise identification of surface defects in semiconductor wafers is critical for controlling manufacturing quality. They develop a multi-scale visual perception network architecture for automated wafer defect pattern recognition. By effectively integrating fine-grained texture cues across resolutions, their approach achieved state-of-the-art accuracy on a real-world industry dataset, demonstrating feasibility for quality inspection.

Self-supervised representation learning from multimodal data. For human action recognition, Yang et al. present a novel framework applying contrastive self-supervised learning on paired unlabeled data (skeleton sequences and inertial sensor signals). Without requiring negative samples, they show superior cross-dataset retrieval and zero-shot transfer performance compared to previous multimodal methods. This highlights the promise of self-supervised techniques to improve model generalization.

Elucidating audiovisual processing in the brain. Understanding the complex neural mechanisms underlying sensory integration remains a key challenge in neuroscience. Jiang et al. combine functional MRI and EEG to construct brain networks involved in audiovisual processing. Through their novel dynamic analysis approach, they revealed early visual-auditory integration occurring prior to attentional effects. These insights shed light on the nature of inter-sensory interactions within the brain.

AI for detecting overloaded trucks to improve road safety. Excessively overloaded trucks pose critical challenges regarding road damage and traffic safety. Sun et al. develop an AI system to detect truck overloading by recognizing truck models from images and matching against weight data. Achieving 85–100% accuracy on small real-world datasets shows feasibility for automated enforcement on highways to improve infrastructure maintenance and prevent hazardous accidents.

More human-like image captioning via reinforced decoding. Generating textual descriptions for images, known as image captioning, requires modeling both visual concepts and language semantics. Bai et al. introduce techniques including guided decoding connections, DenseNets, and reinforcement learning to enhance contextual modeling and feature extraction. Superior results across standard captioning metrics represent tangible progress toward human-level visual understanding.

Targeted smoke reduction to maintain surgical visualization. As discussed by Wang et al., smoke generated during endoscopic procedures can severely obscure surgical sight. They create an enhanced classifier to detect smoke-filled frames prior to selective image enhancement, maximizing efficiency. Achieving high accuracy and speed shows promise for integrated, real-time de-smoking systems to improve situational awareness.

Intelligent product recognition to enable smart vending. As Xu et al. explored, computer vision powered by deep learning can enable emerging autonomous retail models like smart vending machines to accurately recognize products for automatic checkout and inventory status tracking, reducing overhead costs. Their results demonstrate the feasibility of AI to deliver advanced functionality without constant human intervention.

Multi-Scale adaptive learning for robust driving scene parsing. Liu et al. address core challenges in semantic segmentation for autonomous vehicle perception including variations in scale, occlusions and diverse appearances. Their multi-scale adaptive network dynamically selects the most relevant features across levels to accurately parse complex driving environments. State-of-the-art performance on automotive datasets confirms robustness, advancing safety for self-driving systems.

Group-based sparse modeling for image restoration. Recovering high-quality images from incomplete or corrupted inputs remains an active computer vision research area. Ning et al. propose a multi-scale group sparse residual constraint model exploiting patch correlations to effectively eliminate noise and fill in missing regions. Experiments show marked improvements in restoration fidelity compared to existing methods, enabled by joint image priors.

In conclusion, recent advances in visual representation learning could unlock transformative capabilities in transportation, manufacturing, healthcare, and scientific imaging. While progress has been made in tackling real-world vision tasks, continued research into dynamic models, multimodal fusion, and incorporating domain-specific constraints will be instrumental in achieving human-like scene understanding.

## Author contributions

TL: Writing—original draft, Writing—review & editing. QZ: Writing—original draft. FN: Data curation, Writing—original draft. CS: Writing—review & editing.

